# [Retracted] Association between vascular endothelial growth factor gene polymorphisms and the risk of osteonecrosis of the femoral head: Systematic review

**DOI:** 10.3892/br.2024.1814

**Published:** 2024-07-01

**Authors:** Guo Ju Hong, Nuan Lin, Lei Lei Chen, Xiao Bo Chen, Wei He

Biomed Rep 4:92–96, 2016; DOI: 10.3892/br.2015.527

Following the publication of the above article, a concerned reader drew to the Editor’s attention that the authors had referred to a non-existent test (a “Begger’s funnel plot”) in the “*Publication bias*” subsection of the Results (on p. 95).

The authors were asked to comment upon the matter, but the Editorial Office did not receive a reply from them within the requested time-frame for responding. Given that the authors did not choose to reply to address this concern, the Editor has decided that this paper should be retracted from the journal. The Editor also apologizes to the readership for any inconvenience caused.

